# Efficiency of cell-type specific and generic promoters in transducing oxytocin neurons and monitoring their neural activity during lactation

**DOI:** 10.1038/s41598-021-01818-x

**Published:** 2021-11-18

**Authors:** Keerthi Thirtamara Rajamani, Amanda B. Leithead, Michelle Kim, Marie Barbier, Michael Peruggia, Kristi Niblo, Lara Barteczko, Arthur Lefevre, Valery Grinevich, Hala Harony-Nicolas

**Affiliations:** 1 Department of Psychiatry, Icahn School of Medicine at Mount Sinai, 1 Gustave L. Levy Pl, New York, NY 10029 USA; 2 Seaver Autism Center for Research and Treatment, Icahn School of Medicine at Mount Sinai, New York, NY USA; 3 Department of Neuroscience, Icahn School of Medicine at Mount Sinai, New York, NY USA; 4 Friedman Brain Institute, Icahn School of Medicine at Mount Sinai, New York, NY USA; 5 Mindich Child Health and Development Institute, Icahn School of Medicine at Mount Sinai, New York, NY USA; 6 Department of Neuropeptide Research in Psychiatry, Central Institute of Mental Health, Medical Faculty Mannheim, University of Heidelberg, Mannheim, Germany

**Keywords:** Neuroscience, Neural circuits

## Abstract

Hypothalamic oxytocin (OXT) and arginine-vasopressin (AVP) neurons have been at the center of several physiological and behavioral studies. Advances in viral vector biology and the development of transgenic rodent models have allowed for targeted gene expression to study the functions of specific cell populations and brain circuits. In this study, we compared the efficiency of various adeno-associated viral vectors in these cell populations and demonstrated that none of the widely used promoters were, on their own, effective at driving expression of a down-stream fluorescent protein in OXT or AVP neurons. As anticipated, the OXT promoter could efficiently drive gene expression in OXT neurons and this efficiency is solely attributed to the promoter and not the viral serotype. We also report that a dual virus approach using an OXT promoter driven Cre recombinase significantly improved the efficiency of viral transduction in OXT neurons. Finally, we demonstrate the utility of the OXT promoter for conducting functional studies on OXT neurons by using an OXT specific viral system to record neural activity of OXT neurons in lactating female rats across time. We conclude that extreme caution is needed when employing non-neuron-specific viral approaches/promoters to study neural populations within the paraventricular nucleus of the hypothalamus.

## Introduction

The hypothalamus, a brain structure located in the ventral forebrain, plays an essential role in neuroendocrine, autonomic and behavioral regulation^1^. It is composed of several small nuclei, each of which is characterized by various cell types that contribute to physiological and/or behavioral functions^2^. Among these nuclei are the paraventricular nucleus (PVN) and the supraoptic nucleus (SON). Both nuclei contain neurons that produce oxytocin (OXT) or vasopressin (AVP) neuropeptide, which are released from the somatodendritic compartment and/or the axonal terminals of these neurons^3^.

Somatodendritic release allows OXT and AVP to diffuse to hypothalamic and extra-hypothalamic regions^4–6^. Central axonal release from neural terminals allows these peptides to reach extra-hypothalamic brain regions to rapidly modulate behavior, including parenting, pair bonding, social memory, aggression, depression and anxiety, reviewed in^7–10^. For example, limbic and forebrain areas, which are amongst the brain regions activated by social stimuli, as well as brain regions involved in social recognition memory in mice and rats, are mostly innervated by axonal terminals of OXT neurons of the PVN^11–13^ and have been the focus of several functional and behavioral studies^14–19^. Although OXT and AVP are both implicated in autonomic functions and social behaviors, their roles are not identical. Rather they are thought to be complementary, with OXT being more involved in attenuating reactivity to stressful experiences, while AVP is generally associated with arousal and defense, reviewed in^3,9^.

OXT and AVP are also released to the peripheral circulation through their axonal projections to the posterior pituitary gland. AVP is released in response to osmotic stimulation and hemorrhage^20–23^, while OXT is released in response to the stretch of the cervix or stimulation of the nipples during parturition and pup suckling. These stimuli activate OXT neurons and generate a pattern of synchronized burst firing^24–28^, leading to bulk release of OXT into the blood circulation to stimulate uterine contractions or milk letdown^29–31^.

Our knowledge of the neural populations in hypothalamic nuclei and related circuits has immensely expanded as a result of using viral vector-mediated tools and transgenic rodents to achieve targeting of specific neural populations, including OXT and AVP neurons^14–16,18,32–40^. These tools have enhanced our understanding of the anatomical and functional connectivity of OXT and AVP neurons and their respective target brain regions, as well as their specific behavioral and physiological functions^12,14–19,41–48^. Amongst the rodent lines widely used to study OXT neural populations are the OXTp-Ires-Cre-mouse lines and the recently developed OXTp-Cre rat line^37,49^. Other transgenic rat lines, such as the one expressing an OXT- fluorescently tagged fusion protein, OXTp-mRFP1, have also been developed and used to study OXT neuron function^50^. In mouse and rat transgenic lines that expresses a Cre recombinase under the control of the OXT promoter, the expression of the recombinase protein is mostly limited to OXT neurons. When used in combination with viral vectors that carry a transgene, the expression of which is dependent on the activity of the Cre recombinase, these OXTp-Ires-Cre-mouse and the OXTp-Cre rat lines can be employed to specifically label, manipulate, and/or record from OXT neurons^17,19,41–43^. Mouse lines that express a Cre recombinase under the control of the AVP promoter have also been developed and are used in combination with viral tools to study the role of AVP neurons in physiological functions and behaviors^40,44–47^.

Importantly, successful employment of these Cre lines is contingent on the efficacy of the viruses that are used in combination to transduce OXT and/or AVP neurons. To date, there have been no systematic studies comparing the efficacy of different viral serotypes to transduce OXT or AVP neurons, nor has there been a comparison between the efficacies of different non-OXT or AVP promoters in driving the expression of transgenes in these neurons. Such a comparison is important and necessary to identify viral serotypes and promoters that are best suited for transducing OXT neurons and can therefore be used as standalone vectors or in combination with other OXTp-Cre dependent viral vectors or OXTp-Ires-Cre rodent lines. Furthermore, from a gene therapy perspective, achieving targeted expression requires the use of cell-type specific viruses. This is important, particularly in conditions such as neurohypophyseal diabetes insipidus or Prader–Willi syndrome, where there is specific deficiency of AVP and OXT, respectively^51,52^. In this study, we addressed this gap by assessing the efficacy of different adeno-associated viral vectors and several widely used promoters in transducing PVN-OXT and AVP neurons in adult male mice and rats. To demonstrate how these viral constructs can be employed to study OXT neural population in a physiological condition, we used a viral system that expresses a calcium indicator (GCaMP6) under the control of the OXT promoter (AAV1/2-OXTp-GCaMP6s) and recorded neural activity of OXT neurons from the PVN in female rats during lactation. While recording from OXT neurons during lactation has been previously achieved using in vivo electrophysiology methods, this is the first study to record from this specific neural population across days, using a viral vector based approach. This demonstrates the advantage of this tool allowing to record the same OXT neural population from the same animal across time.

## Results

### Transduction of OXT and AVP neurons with different combinations of viral serotypes and promoters in mice and rats

Adeno-associated viral vectors have been widely employed to drive transgene expression in the mammalian central nervous system (CNS)^53,54^. Transduction efficiencies have been reported to vary and are influenced by several factors, including the viral serotype, promoter, cellular subtype (neurons, glia, or oligodendrocytes) and model system (rat, mouse, or non-human primates)^55–58^. Development of newer serotypes and novel pseudo-typing approaches, wherein capsids and genomes from different viral serotypes are combined, has also resulted in improved transduction efficiencies^59,60^. In order to determine the most efficient approach to target OXT and AVP neurons, we first assessed the efficiency of two viral serotypes with known and well-described neuronal transduction efficiencies, AAV1 and AAV9, as well as two pseudotyped viruses, AAVDJ (generated by combining genetic elements from 8 viral capsids)^59,61,62^ and AAV1/2 (containing an AAV1 genome and AAV2 capsid). We combined these serotypes with conventional promoters, namely the human synapsin promoter (SYN), a hybrid promoter (CAG), and a strong constitutively active mammalian promoter, human elongation factor-1 alpha (EF1α). The SYN promoter is highly specific for neurons, while CAG and EF1α are known to drive strong transgene expression across all cell types^63^. Additionally, we included an OXT neuron-specific virus; the AAV1/2-OXTp-Venus^18^, in which the expression of the Venus fluorescent protein is driven by the OXT promoter. Finally, we tested if the Cre recombinase system, driven by the OXT promoter in one virus, can enhance the expression of a floxed-gene that is driven by the CAG promoter in another virus.

#### CAG promoter (AAV1 and AAV9 serotypes)

**Figure 1:**
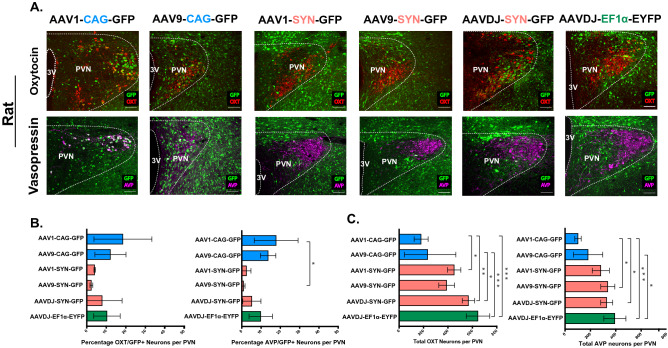
Transduction efficiency of different combinations of adeno-associated viral (AAV) serotypes and promoters in oxytocin (OXT) and arginine-vasopressin (AVP) neurons of the hypothalamic paraventricular nucleus (PVN) of adult rats. (**A**) Confocal images of rat brain tissues, three weeks following viral injection in the PVN and immunostaining with anti-OXT (red, top panel) or anti-AVP (magenta, bottom panel). GFP is encoded by the virus (green, top and bottom panels). 20×, Scale bar = 100 μm, dotted line demarcates the PVN and the 3rd ventricle; 3 V. (**B**) Bar graphs (± SEM) show the average percentage of OXT positive (OXT+, left) or AVP positive (AVP+, right) neurons that also express the virus (GFP+) in the PVN. Low percentages, reflecting low transduction efficiency, were observed across all viruses in both OXT and AVP neurons, with the highest efficiency observed when using AAV serotypes (AAV1 or AAV9) in combination of the CAG promoter. (**C**) Bar graphs (± SEM) show the total number of OXT (left) or AVP (right) neurons per rat PVN. n = 3–4 rats per virus with an average of 10 slices per rat PVN. *p < 0.05, **p < 0.005, ***p < 0.0005.

**Figure 2:**
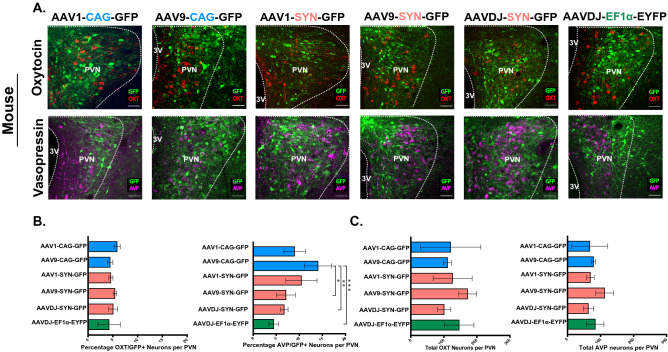
Transduction efficiency of different combinations of adeno-associated viral (AAV) serotypes and promoters in oxytocin (OXT) and vasopressin (AVP) neurons of the hypothalamic paraventricular nucleus (PVN) of adult mice. (**A**) Confocal images of mice brain tissues, three weeks following viral injection in the PVN and immunoblotting with anti-OXT (red, top panel) or anti-AVP (magenta, bottom panel). GFP is encoded by the virus (green, top and bottom panels). 40×, Scale bar = 50 μm, dotted line demarcates the PVN and the 3rd ventricle; 3 V. (**B**) Bar graphs (± SEM) show the average percentage of OXT positive (OXT+, left) or AVP positive (AVP+, right) neurons that also express the virus (GFP+) in the PVN. Low percentages, reflecting low transduction efficiency, were observed across all viruses in both OXT and AVP neurons, with the highest efficiency observed when using the CAG promoter. (**C**) Bar graphs (± SEM) show the total number of OXT (left) or AVP (right) neurons per mouse PVN. n = 3 mice per virus with an average of 5 slices per mouse PVN. *p < 0.05, **p < 0.005, ***p < 0.0005.

We began our analysis by testing the CAG promoter. CAG is a synthetic hybrid promoter, consisting of promoter elements from chicken β-actin promoter fused with enhancer elements from a cytomegalovirus (CMV) and a rabbit β-globin splice acceptor^64^. CAG was previously shown to induce high levels of gene expression across different cell types, including neural populations^65^; therefore, we expected that this promoter would also be efficient in transducing OXT and AVP neurons. For AAV serotypes, we chose to use AAV1 and AAV9, which are both efficient in transducing OXT neurons across the central nervous system (CNS)^61,65^. We found that in rats injected with the AAV1-CAG-GFP or AAV9-CAG-GFP virus, only 18.52 ± 7.41% and 12.12 ± 3.99% of OXT neurons, respectively, and 17.84 ± 5.68% and 13.68 ± 2.0% of AVP neurons, respectively, were also GFP positive (GFP^+^/AVP^+^) (Fig. 1A,B and Table 1). In mice, we found that the infectivity rates were even lower for both viruses. Mice injected with AAV1-CAG-GFP had 5.88 ± 0.36% GFP^+^/OXT^+^ and 8.98 ± 1.33% GFP^+^/AVP^+^ neurons and those injected with AAV9-CAG-GFP had 4.47 ± 0.31% GFP^+^/OXT^+^ and 14.05 ± 1.72% GFP^+^/AVP^+^ neurons (Fig. 2A,B, and Table 2). These findings suggest that the infectivity rate of a CAG promoter, packaged in an AAV1 or AAV9 viral serotype, is similar for OXT and AVP neurons.

**Table 1:** Viral titers and counts for rats.

Virus type	Titer (gc/ml)	OXT count	Fluorophore+/OXT+	%Fluorophore+/OXT+	AVP count	Fluorophore+/AVP+	%Fluorophore+/AVP+
AAV1-CAG-GFP	1.2 × 10^13^	180.5 ± 28.64	34.25 ± 14.01	18.52 ± 7.41	102.75 ± 12.66	19.5 ± 8.25	17.84 ± 5.68
AAV9-CAG-GFP	2.3 × 10^13^	234.5 ± 97.51	25.75 ± 10.77	12.12 ± 3.99	181.75 ± 66.84	28 ± 11.58	13.68 ± 2.0
AAV1-SYN-GFP	1.3 × 10^13^	451.67 ± 31.26	18.33 ± 1.86	4.05 ± 0.22	282.33 ± 38.98	6.33 ± 3.76	2.34 ± 1.24
AAV9-SYN-GFP	1.9 × 10^13^	389.67 ± 37.39	9.33 ± 2.33	2.36 ± 0.42	337.33 ± 31.97	3.33 ± 1.86	0.92 ± 0.44
AAVDJ-SYN-GFP	1.1 × 10^13^	568.33 ± 29.01	43.0 ± 31.0	7.93 ± 5.87	328.67 ± 27.42	18.33 ± 10.41	5.25 ± 2.71
AAVDJ-EF1α-EYFP	4.3 × 10^12^	646.25 ± 47.94	68.25 ± 26.09	10.35 ± 3.39	393 ± 43.49	38.75 ± 14.33	9.83 ± 3.04
AAV1/2-OXT-Venus	1.8 × 10^12^	599.0 ± 15.87	507.0 ± 15.31	84.63 ± 0.6	–	–	–
AAV1/2-SYN-tdTomato	1.2 × 10^11^	336.0 ± 38.39	2.25 ± 0.48	0.74 ± 0.21	–	–	–
AAV1-CAG-FLEX+ AAV1/2-OXT-CRE	1.4 × 10^11^	557.33 ± 34.05	360.67 ± 49.57	64.28 ± 6.32	–	–	–
AAV1-CAG-FLEX+ AAV1-SYN-CRE	5 × 10^13^	266.25 ± 45.04	95.75 ± 9.9	37.51 ± 8.0	–	–	–

**Table 2:** Viral titers and counts for mice.

Virus type	Titer (gc/ml)	OXT count	Fluorophore+/OXT+	%Fluorophore+/OXT+	AVP count	Fluorophore+/AVP+	%Fluorophore+/AVP+
AAV1-CAG-GFP	1.2 × 10^13^	120.67 ± 52.82	7.0 ± 3.0	5.88 ± 0.36	67.33 ± 31.32	6.0 ± 3.0	8.98 ± 1.33
AAV9-CAG-GFP	2.3 × 10^13^	111.33 ± 6.84	5.0. ± 0.58	4.47 ± 0.31	79.0 ± 3.21	11.0 ± 1.0	14.05 ± 1.72
AAV1-SYN-GFP	1.3 × 10^13^	126.67 ± 34.16	5.67 ± 1.20	4.60 ± 0.24	69.33 ± 6.67	7.0 ± 0.58	10.46 ± 1.95
AAV9-SYN-GFP	1.9 × 10^13^	172.33 ± 15.65	9.33 ± 0.67	5.44 ± 0.20	112.67 ± 15.88	7.67 ± 0.33	7.11 ± 1.16
AAVDJ-SYN-GFP	1.1 × 10^13^	101.0 ± 11.53	5.0 ± 0.0	5.07 ± 0.52	63.33 ± 8.99	4.33 ± 0.88	6.74 ± 0.48
AAVDJ-EF1α-EYFP	4.3*10^12^	146.67 ± 25.67	5.67 ± 1.20	4.25 ± 1.31	84.33 ± 14.99	3.67 ± 1.15	4.45 ± 1.03
AAV1/2-OXT-Venus	1.8 × 10^12^	124.0 ± 14.01	99.33 ± 13.30	80.16 ± 5.56	–	–	–
AAV1/2-SYN-tdTomato	1.2 × 10^11^	148.0 ± 4.0	2.0 ± 1.0	1.33 ± 0.94	–	–	–

#### SYN promoter (AAV1, AAV9, and AAVDJ serotypes)

To examine if the low transduction efficiency of OXT and AVP neurons was attributed to the CAG promoter or to the viral serotypes (AAV1 and AAV9), we chose to examine if the transduction rate improves by using a neuron-specific promoter, SYN, in combination with the same viral serotypes (AAV1 and AAV9) or with a third viral serotype such as the AAVDJ, which is a hybrid capsid derived from 8 different serotype*s,* while using the same SYN promoter. To our surprise, we found that the transduction rates using AAV1-SYN AND AAV9-SYN were very low. In rats injected with AAV1-SYN-GFP, only 4.05 ± 0.22% and 2.46 ± 1.33% of the neurons were GFP^+^/OXT^+^ and GFP^+^/AVP^+^, respectively, and in rats injected with AAV9-SYN-GFP, only 2.36 ± 0.42% and 0.92 ± 0.44% of the neurons were GFP^+^/OXT^+^ and GFP^+^/AVP^+^, respectively (Fig. 1A,B, and Table 1). In mice injected with AAV1-SYN-GFP only 4.60 ± 0.24% and 10.46 ± 1.95% of the neurons were GFP^+^/OXT^+^ and GFP^+^/AVP^+^, respectively, and in those injected with AAV9-SYN-GFP, only 5.44 ± 0.20% and 7.11 ± 1.16% of the neurons were GFP^+^/OXT^+^ and GFP^+^/AVP^+^, respectively (Fig. 2A,B, and Table 2). Furthermore, rats and mice injected with the AAVDJ-SYN-GFP virus did not show improved transduction rates when compared to other serotype/promoter combinations. In rats, only 7.93 ± 5.87% and 5.25 ± 2.71% of the neurons were GFP^+^/OXT^+^ and GFP^+^/AVP^+^, respectively (Fig. 1A,B, and Table 1) and in mice, only 5.07 ± 0.52% and 6.74 + 0.48% of the neurons were GFP^+^/OXT^+^ and GFP^+^/AVP^+^, respectively (Fig. 2A,B and Table 1).

#### Ef1α promoter (AAVDJ serotype)

We next tested the mammalian ubiquitous promoter, Ef1α. Despite the wide use of this combination of the *AAVDJ* viral serotype and the Ef1α promoter in the CNS^66–68^ *,* the infection rates of OXT neurons were still very low in both rats and mice. In rats injected with AAVDJ-EF1α-EYFP, we found that 10.35 + 3.39% and 9.83 + 3.04% of neurons were EYFP^+^/OXT^+^ and EYFP^+^/AVP^+^, respectively (Fig. 1A,B and Table 1) and in mice, 4.25 ± 1.31% and 4.45 ± 1.03% of neurons were EYFP^+^/OXT^+^ and EYFP^+^/AVP^+^, respectively (Fig. 2A,B and Table 2).

#### Comparison between viruses

Overall, our findings show no major differences in the efficiency of the viruses tested above to transduce OXT or AVP neurons. However, we did observe higher transduction efficiency for the AAV9-CAG virus in AVP neurons in mice, compared to viruses that carry the SYN and EF1α promoters (One-way analysis of variance (ANOVA), *F*_5,12_ = 6.43, *p* = 0.004; Tukey post-hoc analysis: AAV9-CAG vs AAV9-SYN, *p* = 0.027; AAV9-CAG vs AAVDJ-SYN, *p* = 0.020; AAV9-CAG vs AAV-DJ-EF1α, *p* = 0.003). Moreover, we found that the total number of OXT and AVP neurons per PVN did not differ significantly across rats or mice that were injected with any of the viruses (Figs. 1C, 2C and Tables 1 and 2), with the exception of AAV1-CAG-GFP or AAV9-CAG-GFP in rats. Rats injected with either of these viruses had lower number of OXT neurons in the PVN (Fig. 1C and Table 1) (ANOVA *F*_5,15_ = 11.20, ***p = 0.0001; Tukey post-hoc analysis: AAV1-CAG vs AAV1-SYN, *p = 0.043; AAV1-CAG vs AAV-DJ-SYN, **p = 0.003; AAV1-CAG vs AAVDJ-EF1α, **p < 0.001; AAV9-CAG vs AAVDJ-SYN, *p = 0.01; AAV9-CAG vs AAVDJ-EF1α, ***p < 0.001) and AVP neurons in the PVN (ANOVA *F*_5,15_ = 8.06, *p* = 0.0007; AAV1-CAG vs AAV9-SYN, *p = 0.01; AAV1-CAG vs AAV-DJ-SYN, *p = 0.014; AAV1-CAG vs AAV-DJ-EF1α, ***p < 0.001; AAV9-CAG vs AAV-DJ-EF1α, *p = 0.013).

#### OXT promoter (AAV1/2 serotype)

**Figure 3:**
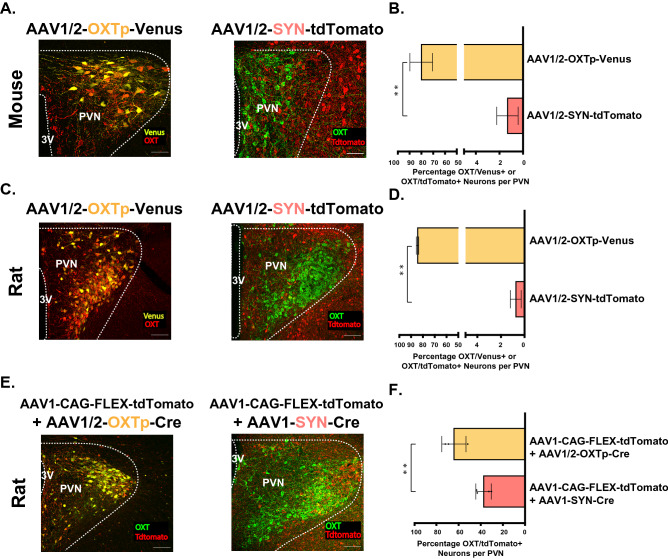
OXT promoter and not viral serotype confers high degree of specificity and transducibility for viruses targeting OXT neurons. (**A**) Confocal images of mice brain tissues, three weeks following viral injection in the PVN. Left, injection with AAV1/2-OXTp-Venus, immunoblotting with anti-OXT (red) and Venus is encoded by the virus (yellow). Right, injection with AAV1/2-OXTp-tdTomato, immunoblotting with anti-OXT (green) and tdTomato is encoded by the virus (red). 40×, Scale bar = 50 μm, dotted line demarcates the PVN and the 3rd ventricle; 3 V. (**B**) Bar graphs (± SEM) show the average percentage of OXT positive (OXT+) neurons that also express the virus (Venus^+^ or tdTomato^+^) in the PVN. Transduction efficiency is significantly higher for the AAV1/2-OXT-Venus virus. (**C,D**) Same as **A** and **B**, respectively, but in rats. Transduction efficiency is significantly higher for the AAV1/2-OXT-Venus virus. Transduction efficiency is significantly higher for the AAV1/2-OXT-Venus virus. (**E**) Confocal images of mice brain tissues, three weeks following viral injection in the PVN. Left, injection with AAV1/2-OXT-Cre + AAV1-CAG-FLEX-tdTomato. Right, injection with AAV1-CAG-Flex-tdTomato and AAV1-SYN-CRE. In both images, immunoblotting with anti-OXT (green) and tdTomato is encoded by the virus (red). (**F**) Bar graphs (± SEM) show the average percentage of OXT positive (OXT+) neurons that also express the virus (Venus^+^ or tdTomato^+^) in the PVN. Transduction efficiency is significantly higher for the AAV1/2-OXT-Venus + AAV1-CAG-FLEX-tdTomato combination. 40×, Scale bar = 50 μm, dotted line demarcates the PVN and the 3rd ventricle; 3 V. n = 3 rat per virus with an average of 10 slices per rat PVN. *p < 0.05, **p < 0.005, ***p < 0.0005.

Since the OXT promoter had been already characterized^69–72^ and used previously to specifically drive the expression of different genes, including Cre, Venus^18^, and GCaMP6s^33^ in OXT neurons, we decided to include the virus that expresses an OXT promoter-driven fluorescent protein as a positive control for our analysis: the AAV1/2-OXTp-Venus^18^. As expected, and as has been previously reported, this virus showed a very high transduction rate in the OXT neurons of both rats and mice^15,18^. We found that 80.16 ± 5.56% and 84.63 ± 0.60% of neurons were Venus^+^/OXT^+^ in mice (Fig. 3A—left panel, B and Table 2) and rats (Fig. 3C—left panel, D and Table 1), respectively. To confirm that the high transduction rate is mostly attributable to the OXT promoter and not to the AAV1/2 serotype, we examined an additional virus with the same serotype but a different promoter, the AAV1/2-SYN-tdTomato. We found that in both mice (Fig. 3A—right panel, B and Table 2) and rats (Fig. 3C—right panel, D and Table 1) the transduction efficiency of this virus was significantly lower than the AAV-OXTp-Venus, with only 1.33 ± 0.94% of mice and 0.75 ± 0.23% of rat OXT neurons being tdTomato^+^/OXT^+^ (Mice, Unpaired Student’s *t*-test, two-tailed *t*(3) = 10.95; **p = 0.002); Rats *t*(5) = 146.7, **p < 0.0001). Together, these findings confirm that the high transduction rate of OXT neurons is mostly attributed to the OXT promoter.

#### OXT promoter in combination with a Cre recombinase system

The employment of OXTp-Ires-Cre mouse and rat lines^15,18,37,49^ and the use of OXTp-Cre containing viruses to drive specific gene expression in OXT neurons necessitates the use of an additional virus that carries that gene and is dependent on the activity of the Cre recombinase to drive expression. Therefore, we next asked if a combination of a virus that expresses a Cre recombinase under the control of OXT promoter can overcome the caveats we observed in using non-OXT specific promoters. To address this question, we tested and compared the efficacy of each of the following viral combination in transducing OXT neurons in rats: (1) AAV1-CAG-FLEX-tdTomato + AAV1/2-OXTp-Cre and (2) AAV1-CAG-FLEX-tdTomato + AAV1-SYN-Cre, the latter of which serves as a control that does not include the OXT promoter. We found that driving the Cre expression under the OXT promoter, using the first combination, led to a significantly higher transduction rate, 64.28 ± 6.32%, as compared to that of the second combination; 37.51 ± 3.48% (Unpaired Student’s *t*-test, two-tailed *t*(5) = 3.99; **p = 0.01; Fig. 3E,F and Table 1). Together, these findings suggest the transduction rate of viruses in OXT neurons can be significantly improved when combined with an OXT promoter-driven Cre system.

### OXT neural activity in lactating rats

**Figure 4:**
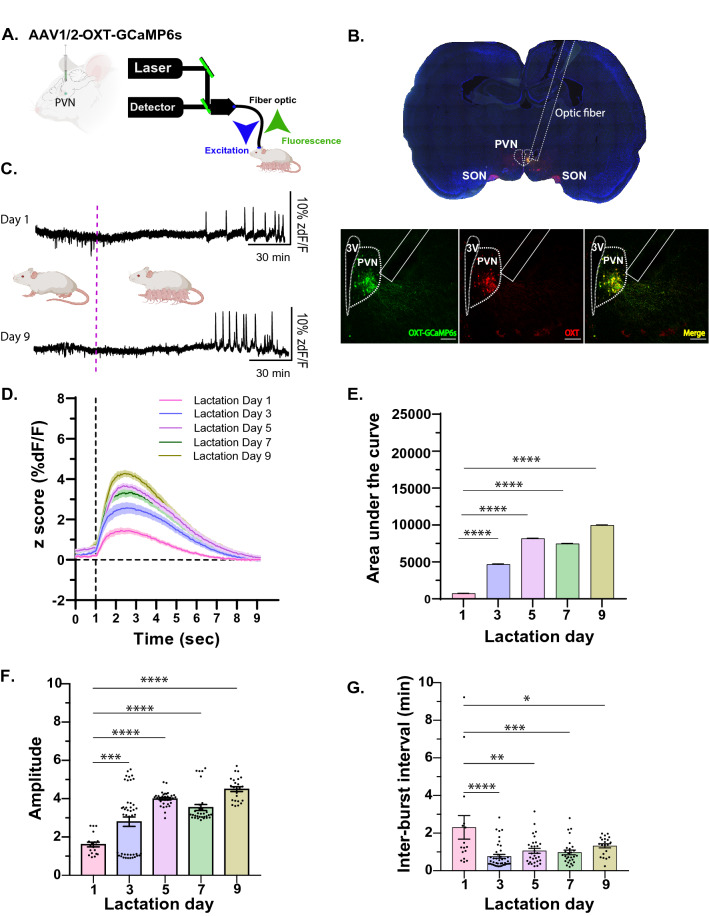
Amplitude and frequency of OXT neural response dynamics over time and across days of lactation. (**A**) A schematic showing viral injection of the AAV1/2-OXTp-GCaMP6s viral injection and the fiber photometry set up. (**B**) Tiled image (top) of a rat coronal section shows the position of the optic fiber relative to OXT neurons within the PVN. Bottom image shows GCaMP6s (left, green), whose expression is under the control of the OXT promoter, OXT labeling using anti-OXT antibodies (middle, red), and the overlap between the two (right, yellow), demonstrating the specificity of the virus. Scale bar = 100 μm. (**C**) Representative traces of OXT neurons activity (%zdF/F), recorded on days 1, 5, 7 and 9 of lactation from the PVN of lactating rat. Dotted magenta line indicates when pups were re-introduced to the lactating rat. (**D**) Average lactation-dependent GCaMP6s responses of PVN-OXT neurons across days. Each day represents average responses from 3 female rats during lactation (10–17 bursts per rat). (**E**) Area under the curve was calculated from average lactation-dependent responses across days. (**F**) Amplitude of lactation-dependent response increases over lactation days. (**G**) Inter-burst intervals of lactation-dependent response, is highest on day 1 of lactation (Day 1), reflecting lower frequency and increases on Day 3, reflecting an increase in frequency, which remains consistently the same across consequent days of lactation. 3 V, 3rd ventricle, PVN, Paraventricular Nucleus; SON, Supraoptic Nucleus. Z score (zdF/F), z scored change in GCaMP6s-dependent response over non-GCaMP6s-dependent response. *p < 0.05, **p < 0.005, ***p < 0.0005. Part of **A** and **C** were created with https://BioRender.com.

To demonstrate the utility of the OXT promoter for conducting functional studies, we used the AAV1/2-OXTp-GCaMP6s^33^, which expresses a calcium indicator, (GcaMP6)^73^ under the control of the OXT promoter and a fiber photometry system, to record neural activity of OXT neurons in female rats during lactation. Specifically, we injected the AAV1/2-OXTp-GCaMP6s virus into the PVN of female rats and implanted an optical fiber in the same region to deliver a blue light at 465 nm wavelength. The same optic fiber also captures the emitted green light resulting from a conformational change in the green fluorescent protein that is tagged to the calcium indicator resulting from elevated calcium activity, which serves as a proxy for neural activity (Fig. 4A,B). One week after recovery from surgery, female rats were mated with male rats to induce pregnancy, delivery and eventually lactation. Using the fiber photometry approach, we recorded burst activity of OXT neurons during lactation across several days: Day (d) 1, 3, 5, 7, and 9 (Fig. 4C–E). We found an overall effect of time on the magnitude of GCaMP6s responses in OXT neurons during lactation (Fig. 4D,E) (One-way ANOVA, *F*_4, 45775_ = 6509, *p* < 0.0001). Post-hoc analysis (Dunnett’s multiple comparisons test) revealed a statistically significant difference in the magnitudes across days, relative to d1 (day d1 vs. d3, ***p < 0.0001; d1 vs. d5, ****p < 0.0001; d1 vs. d7, ****p < 0.0001; d1 vs. d9, ****p < 0.0001. Furthermore, we found a statistically significant effect of time on the amplitude of the GCaMP6s responses (Fig. 4F) (One-way ANOVA, *F*_4,146_ = 27.49, ****p < 0.0001). Post-hoc analysis (Dunnett’s multiple comparisons test) revealed a significant increase in amplitude relative to d1 (d1 vs. d3, ****p = 0.0002; d1 vs d5, ****p < 0.0001; d1 vs. d7, ****p < 0.0001; d1 vs. d9, ****p < 0.0001). By measuring the time between each individual burst (frequency) across each recording session and comparing it across days (d1 through d9), we found a significant effect of time on frequency (One-way ANOVA, F_4,131_ = 6.780, *p* < 0.0001) (Fig. 4G, inter-burst interval). Mainly, we observed that the frequency of burst firing was lower on d1 (higher inter-burst interval), relative to the remaining recording sessions (d1 vs d3, ****p < 0.0001; d1 vs. d5, **p = 0.0015; d1 vs. d7, ***p = 0.007; *d1 vs. d9, *p* = 0.04). The frequency of bursts, however, remained consistently the same through d3 and d9.

These findings provide, for the first time, in vivo recording data from lactating female rats across post-natal days. Our findings also agree with previous studies that use mammary pressure as a proxy for milk ejection and OXT neural activity, which reported increases in the amplitude of milk ejection responses to exogenous oxytocin during late pregnancy and lactation^74^.

## Discussion

The use of recombinant adeno-associated viruses to target specific neural populations has contributed significantly to our understanding of their unique roles in modulating physiology and behavior. This has been aided greatly by the development and use of genetically engineered tools such as DREADDs (designer receptors activated by designer drugs), opsins and genetically encoded calcium indicators (GECIs). These tools have been used to target discrete neural subsets through the use of viral vectors with particular selectivity for cell types and/or the use of gene specific promoter elements^75^. Although several AAVs have been widely used in the CNS, the cellular tropism and efficiencies vary between serotypes and depend on the targeted brain region and/or cell type^61,76–78^.

The hypothalamus is a highly diverse structure with at least 34 different neuronal and 11 non-neuronal subtypes identified thus far^79^. This heterogeneity presents a need for targeting specific neuronal subpopulations to study the unique contributions of each of these cell types. As such, investigating these neural populations requires tools and strategies that ensure efficiency and specificity at targeting. Several strategies, including the development of transgenic mouse lines using the Cre-lox system, have been employed to study the role of OXT and AVP neurons in behavior and physiology^14,15^. The emergence of genome editing tools including CRISPR-Cas9 has accelerated the development of gene-specific Cre-driver rat lines, although only a few of these lines are readily available^37,49,80^. Furthermore, employing virogenetic tools like these in higher organisms including non-human primates, is impractical for ethical and financial reasons. As such, we remain heavily reliant on the use of viral tools and gene specific promoters to drive transgene expression in a cell type-specific manner^81–83^. In this study, we evaluated transduction efficiencies of four different viral serotypes (AAV1, AAV9, AAVDJ, and AAV1/2) and three widely used promoters (CAG, SYN, and Ef1α) in targeting OXT and AVP neurons in mice and rats. To our surprise, we found that when used in combination with native or synthetic promoters, all four serotypes had low transduction efficiencies (~< 30%) in both mice and rats.

The human SYN promoter was first cloned in 1989 and has long been demonstrated to express in all neurons and has been widely employed to drive transgene expression in almost every neuronal subtype^84^. Our findings that this promoter was not efficient at transducing OXT or AVP neurons, regardless of the serotype combination, suggests the SYN promoter activity is fairly weak in this population of neurons. This low level of SYN1 promoter activity could be reflective of the low expression level of Synapsin 1 in the paraventricular nucleus of the hypothalamus^85^ and its low expression in OXT and AVP neurons^86^. This could potentially be attributed to the fact that, although OXT neurons send extensive projections to extrahypothalamic regions, they rarely form classical synapses^12,18,87^. Given that mammalian promoters are generally considered as weak activators of transcription, we used a strong hybrid synthetic promoter, CAG, which combines elements derived from chicken β-actin promoter and the CMV promoter (derived from the virus, Cytomegalovirus). Although we did find a marginal improvement in transduction efficiencies, particularly with AAV1-CAG serotype (rats) and AAV9-CAG serotype (mice) in infecting AVP neurons, we also observed significantly lower number of total OXT and AVP neurons (rats) when using either AAV1-CAG or AAV9-CAG. This decrease in total number of neurons could be also the result of cellular toxicity, induced by excessive promoter activity^88^, which could have inflated the transduction efficiency rate. As such, lowering the titer when using strong constitutive promoters may be necessary to mitigate effects of viral-mediated toxicity.

In addition to gene-specific promoters, various hybrid vectors have also been engineered to increase transduction efficiency^89^. AAVDJ is a variant generated from a library of eight different AAV wild type serotypes^90^ and has been widely employed to deliver transgenes to the CNS^68^. Surprisingly, we found that the DJ variant, when combined with a native (SYN) or a strong mammalian promoter (EF1α), produced no greater improvement in transduction efficiencies compared to other serotype/promoter combinations. Given that none of the tested native or constitutively active hybrid promoters/viral vector combinations were useful at transducing OXT or AVP neurons, we tested how gene specific promoters fared at targeting OXT neurons. For this part of the study, we chose to focus on OXT, as extensive work has been done in identifying upstream transcription elements in the OXT promoter that regulate cell-type specific expression, including the identification of minimum upstream promoter regions required for conferring specificity^39,91,92^. Our results demonstrate that 1.9 kb OXT promoter, selected by the homology of sequence upstream to ATG between mammalian species^18^, is optimal for effective transduction of OXT neurons at very high rates (> 80%) and that viral serotypes have very little contribution in driving this high transduction rate. It also suggests that high transduction efficiency using the AAV/12-OXTp-Venus virus could be due to the high activity (strength) of the OXT promoter leading to increased expression of the downstream gene. It is important to note, however, that our findings do not rule out the possibility that different combinations of OXT promoter and viral serotypes can influence transduction. For example, Fields and colleagues have shown that specific and effective targeting of OXT neurons can be achieved using a different OXT promoter sequence, further confirming our findings, and have also demonstrated that different AAV serotypes in combination with the OXT promoter may produce varying rates of transduction^92^. Taken together with our findings, this suggests that using a neuron specific promoter is the most efficient approach to transduce OXT neurons and that the efficiency can be further optimized by combining the OXT promoter elements with specific viral serotypes.

In the absence of Cre-driver mouse or rat lines to target specific cell populations, a dual virus approach can be used to target cell-specific expression. Using this approach, one virus expresses a Cre recombinase and the other expresses a gene that is Cre-dependent (e.g. cloned in between two loxP sites). Therefore, in this study we set to test to what extent the use of a gene specific promoter enhances the transduction efficiency using the Cre-floxed system. We found that the combination of an OXTp-Ires-cre with a cre-dependent virus (AAV1-CAG-FLEX), increased the transduction efficiency by ~ 0.7 fold. These findings suggest that when employing either a one- or two-virus approach, the use of a gene-specific promoter dramatically enhances the transduction of OXT neurons.

The development of novel virogenetic tools (DREADDs, optogenetics and GECI’s) has opened the door to test the specific role of neuropeptides in a variety of behaviors. Combining these tools with gene-specific promoters exponentially changes our ability attribute specific cell populations to distinct physiological behaviors^33^. To that extent, we recorded activity of OXT neurons across several days during lactation using fiber photometry. Although single cell OXT response to suckling has been previously recorded using intracellular recordings on slice preparations, organotypic slices or *in vivo*^93,94^, this is the first study to follow OXT-PVN neural responses in lactating rat females across days, using viral tools in combination with fiber photometry. Sutherland and colleagues recorded milk ejection in pregnant and nursing rats and the response to OXT administration, showing that milk ejection (as a function of change in intramammillary pressure) increases during pregnancy but not during lactation^74^. In our recordings, we found that OXT neurons change their responses to suckling over time, wherein higher amplitudes of OXT bursts were recorded across days. On the other hand, the frequency of OXT dependent neural responses remained lower on the first day of testing but gradually increased as the pups got older. The change in frequency and amplitudes can be attributed to increased suckling pressure over time as the pups get older and the demand for nutrition increases over time. However, it is unclear if and how these changes in frequency and amplitude of OXT bursts temporally correlate with increase in milk let-down or intramammillary pressure. Ideally, a greater degree of temporal precision can be achieved if intramammillary pressure and OXT neuron firing can be recorded simultaneously using our model. Thus, by combining a gene specific promoter with novel genetic tools we demonstrated for the first time their usefulness in studying physiological behaviors such as lactation across time.

Finally, we acknowledge it is not possible to test every available serotype/promoter combination and it is likely that other serotypes and/or promoter combinations have a greater ability to transduce OXT and AVP neurons^95,96^. Furthermore, it is also important to note that gene-specific promoters are far from perfect as recently demonstrated by Kakava-Georgiadou et al.^75^. Alternatively, engineering heterologous minimal promoters with their enhancers is a promising new approach towards cell-type specific tagging of specific neuronal types^97^. It is also important to determine optimal experimental conditions (such as viral titer, volume, stereotaxic coordinates and the time of viral expression) even when employing available “standard” cell-type specific promoters. Taken together, we suggest extreme caution in choosing the right viral vector approach to study neural populations within the PVN, as getting the right combination goes a long way in achieving targeted gene expression.

## Materials and methods

### Animals

We used adult male Sprague Dawley rats (Charles River, Wilmington, MA, USA) and adult male C57BL/6T mice (Taconic Biosciences, Germantown, NY, USA). All stereotaxic injections were done at the age of 8–10 weeks. Animals were housed in groups of 2 (rats) or 2–5 (mice) under a 12 h light/dark cycle at 22 ± 2 °C with food and water available ad libitum. All animal procedures were carried out in accordance with protocols approved by the Institutional Animal Care and Use Committee at the Icahn School of Medicine at Mount Sinai. The study was conducted in compliance with ARRIVE guidelines.

### Viral vectors

We tested twelve adeno associated viral vectors (AAV) for their ability to transduce oxytocin and arginine-vasopressin neurons in the PVN: AAV-CAG-GFP and AAV-SYN-GFP, serotypes 1 and 9 (catalog numbers: 37825 and 50465, respectively, Addgene, Cambridge, MA, USA); AAVDJ-SYN-GFP and AAVDJ-Ef1α-EYFP (catalog numbers: GVVC-AAV-127 and GVVC-AAV-168, respectively, Stanford Viral and Vector core, Stanford, CA, USA); AAV1-CAG-FLEX-tdTomato and AAV1-SYN-HI-eGFP-CRE-WPRE-SV40 (catalog numbers: 28306 and 105540, respectively, Addgene, Cambridge, MA, USA); AAV1/2-OXTp-Venus, AAV1/2-OXTp-Ires-CRE and AAV1/2-SYN-tdTomato and AAV1/2-OXTp-GCaMP6s produced by Dr. Valery Grinevich’s lab at the University Heidelberg, Germany. Viral titers are detailed in Tables 1 and 2.

### Stereotaxic surgery for viral injection

Animals were anesthetized with 3–5% isoflurane for induction and then isoflurane was maintained at 1.5–2.5% with 2% oxygen using a tabletop vaporizer and a non-breathing circuit. The surgical area was shaved, aseptically cleaned and an incision was made along the dorsal midline of the skull. After clearing the connective tissue, bregma and lambda were identified, the region of injection was marked, and a small burr hole (50 μm) was drilled. For rats, the virus was loaded into a 10 μl Hamilton syringe (Hamilton and company, Las Vegas, NV, USA) and 0.3 μl of the virus was injected into the PVN at a 10° angle (A–P − 1.7 mm, M–L 0.3 mm, D–V 8.0 mm) at a rate of 0.1 μl/min. For mice, the virus was loaded into a 10 μl 33G NanoFil syringe (World Precision Instruments, Sarasota, FL, USA) and 0.3 μl of the virus was injected into the PVN at a 10° angle (A–P − 0.6 mm, M–L+ − 1.0 mm, D–V 5.15 mm) at a rate of 0.1 μl/min. Following the injection, the syringe was left in place for 10 min and withdrawn at a rate of 0.2 mm/min. Incision wound was closed using wound clips for rats (EZ Clips, Stoelting Inc, Wood Dale, IL, USA) or sutured in mice (Ethilon Suture 5-0, Henry Schein, Melville, NY, USA). Rodents received intraoperative subcutaneous fluids for hydration (Lactated Ringer Solution, Thermo Fisher Scientific, Waltham, MA, USA) and buprenorphine (0.5 mg/kg) for analgesia. Additional analgesia was administered subcutaneously every 12 h for 72 h post-operatively.

### Histology

Rats were anesthetized with an intraperitoneal injection of Ketamine (100 mg/kg) and Xylazine (13 mg/kg) and mice were anesthetized with 3–5% isoflurane for induction. Once a surgical plane of anesthesia was achieved, rats were peristaltically perfused at a rate of 40 ml/min with 0.2 M Sodium Phosphate Buffer for 2–3 min followed by 4% paraformaldehyde (PFA) for 20 min. Mice were perfused with 0.2 M Sodium Phosphate Buffer followed by 4% PFA at a rate of 8 ml/min for 10 min. Brains were removed, immersed in 4% PFA overnight at 4 °C, then placed in a sucrose solution (30% sucrose, 100 mM glycine and 0.05% sodium azide in 1×PBS) for 48 h. Brains were frozen in a cryomold filled with O.C.T. (Tissue-Tek, Torrance, CA) and stored at − 80 °C until sectioning with a cryostat (Leica CM 1860 Leica Biosytems, Buffalo Grove, IL, USA).

### Immunohistochemistry

Brain sections (40 μm) including the paraventricular nuclei (PVN) region (bregma − 0.6 to − 2.0 mm Anterior–Posterior (A–P) in rats, bregma − 0.6 to 1.2 mm A–P in mice) were collected and alternate sections were designated for OXT or AVP staining. A total of 9–10 (rats) and 4–6 (mice) sections spanning the entire PVN were stained for either OXT or AVP. Sections were washed (3 × 10 min each in 1×PBS, 0.05% Triton X-100), blocked and permeabilized for 1 h in 5% donkey serum (Jackson ImmunoResearch, West Grove, PA, USA), 0.5% Triton X-100 in 1×PBS and stained with anti-oxytocin PS38 mouse monoclonal antibody or anti-vasopressin PS41 mouse monoclonal antibody (a gift from Dr Harold Gainer, NIH, Bethesda, USA)^98^ (1:500 in 5% donkey serum and 0.5% Triton X-100 in PBS) then left overnight at 4 °C. The following day, sections were washed and incubated in Alexa Fluor 594 Donkey anti-Mouse IgG or Alexa Fluor 488 Donkey anti-Mouse IgG (for OXT-Cre, AAV1-SYN-CRE, and AAV1/2-SYN-tdTomato experiments) (1:1000 in 0.5% Triton X-100 in PBS; cat. no. A-21203; ThermoFisher Scientific, MA, USA) for 1 h at room temperature. Sections were then washed and mounted with VECTASHIELD Antifade Mounting Medium with DAPI (cat. no. H-1200, Vector Labs, Burlingame, CA, USA).

### Microscopy and image analysis

Briefly, PVN slices were imaged on a fluorescent microscope (EVOS FL Auto 2, ThermoFisher Scientific, MA, USA). Z-stack images were acquired at step size of 1.5 μm and OXT and AVP-immunoreactive neurons were counted manually using the ImageJ software^99^. Briefly, grid settings were applied to an RGB image and the point tool used to count stained OXT/AVP and GFP, Venus, or tdTomato positive neurons. In both rats and mice, AVP signal was pseudo colored to magenta. Overlap of GFP, Venus, and tdTomato with either OXT or AVP stained neurons was visually determined and quantified as (number of GFP^+^, Venus^+^, or tdTomato^+^ neurons)/(OXT^+^ or AVP^+^ neurons) / total number of OXT or AVP neurons * 100. Confocal microscopy was performed at the Microscopy CoRE at the Icahn School of Medicine at Mount Sinai. Images were acquired using Leica SP5 DMI at 20× and 40× (oil) magnification for rat and mouse tissue respectively. Z stacks were acquired at step size of 1.5 μm (20×, rat) and 1.0 μm (40×, mice). Stacked images were exported to FIJI (FIJI is just ImageJ)^99^ and single plane images were generated using Z project (maximum intensity projection). For fiber photometry, a 10× image of the brain section was acquired on a Leica dm18 and subsequently tiled, using FIJI software.

### Lactation induced fiber photometry recording

8 week old sexually mature rats were injected unilaterally with AAV1/2-OXT-GCaMP6s in the PVN (A–P, 1.5 mm, M–L, 0.3 mm, D–V, 7.8 mm at a 15° angle). Immediately after, a fiber optic cannula (400 μm 0.39NA, Cat. CFM14L10, Thor Labs, Newton, New Jersey) was implanted in the same brain region. One week later, the injected and implanted female rat was housed with a single male for mating. Pregnancy was monitored and day of birth was noted as day 0. 24 h following birth, female animal along with its litter was transported to the behavior room. All pups (except for 1 that remained with the mother) were separated from the mother for 3 h during which they were maintained at 37 °C using a heating pad. 15 min prior to introduction of the pups, a 5 min fiber photometry response was recorded from the female by connecting a fiber optic patch cord (400 μm, 0.48NA, Doric lenses, Quebec, Canada) to the fiber optic cannula. The remaining pups were reintroduced into the cage containing the female and recording continued for 45 min. The litter size (11 pups) was kept consistent between females. Fiber photometry recordings on alternate days and during the same time of the day. Fiber photometry responses were recorded from 3 different animals.

### Fiber photometry data analysis

Demodulated signal was acquired using a TDT microprocessor (Tucker-Davis Technologies, FL, USA). Briefly, 465 and 405 nm LED were driven at 400 mA and 200 mA respectively, with the power at the tip of the fiber optic cannula determined to be at 80–120 uW. Data was extracted using a modified Matlab script based on published work^100^. Briefly, 465 and 405 signals were extracted and smoothed using a moving mean algorithm. This was followed by baseline correction of the two signals using airwighted adaptive iteratively reweighted Penalized Least Squares (airPLS) algorithm (https://github.com/zmzhang/airPLS). Each signal was then independently standardized and the standardized 405 (std405) signal was fitted to the standardized 465 (std465) signal using a non-robust linear regression function. Finally, normalized zdF/F was calculated using the formula, zDF/F = zscore(std465)-zscore(std405). %zdF/F for each lactating event was extracted and averaged across all animals for each day of recording. The area under the curve for the averaged %zdF/F was calculated using GraphPad software (GraphPad Prism, San Diego, CA, USA) using the area under the curve function. Amplitude was calculated using the findpeaks function on Matlab (Mathworks, Natick, MA, USA) with minimum peak distance of 0.2 and minimum peak height of 1.0. Inter burst interval was calculated by computing the difference in time between two adjacent bursts.

### Statistical analysis

For comparing total OXT and AVP counts and percentage overlap, a one-way ANOVA (analysis of variance) was used. Group means were compared using multiple comparisons and adjusted using Tukey test. Unpaired two-tailed Student’s *t*-test was used to compare transduction efficiencies of the OXT promoters. Lactation based GCaMP6 responses (area under the curve, amplitude and frequency) were analyzed by one-way ANOVA followed by post-hoc analysis using Dunnett’s multiple comparisons test.
